# Twenty‐Two Years of Shrub Encroachment and Its Effects on Bird Communities in an African Savanna

**DOI:** 10.1002/ece3.72594

**Published:** 2025-12-02

**Authors:** Muzi D. Sibiya, Wisdom M. Dlamini, Robert A. McCleery, Clelia Sirami, Ara Monadjem, Robert J. Fletcher

**Affiliations:** ^1^ School of Natural Resources and Environment University of Florida Gainesville Florida USA; ^2^ Department of Biological Sciences University of Eswatini Kwaluseni Eswatini; ^3^ Department of Wildlife Ecology and Conservation University of Florida Gainesville Florida USA; ^4^ Department of Geography University of Eswatini Kwaluseni Eswatini; ^5^ Dynafor, Université de Toulouse, INRAE, INPT, INP‐EI Purpan Castanet Tolosan France; ^6^ Mammal Research Institute, Department of Zoology & Entomology University of Pretoria Hatfield South Africa; ^7^ Department of Zoology, Conservation Research Institute University of Cambridge Cambridge UK

**Keywords:** birds, occupancy, savanna, shrub encroachment, trait, woody

## Abstract

Open terrestrial ecosystems such as savannas have been experiencing marked increases in woody cover driven by shrub encroachment. Despite this widespread pattern, understanding the consequences for faunal communities remains challenging because long‐term data are often not available and other structural changes, such as changing tree cover, may confound conclusions on shrub encroachment effects. We used satellite data and surveys of bird communities spanning 22 years to assess vegetation‐cover dynamics and its effects on bird communities across the savanna ecosystem of Eswatini. We employed a hierarchical multi‐species occupancy model that accounted for imperfect detection to assess changes in species occurrence, richness, and community assemblages. Between 1998 and 2020, shrub cover increased from 16% to 44% and tree cover increased from 17% to 28%. Across 64 species, shrub cover tended to have greater effects on bird occupancy than tree cover, with 34 (53%) species exhibiting positive linear associations with shrub cover and 15 (23.4%) species exhibiting a non‐linear response to shrubs, where occupancy peaked at < 50% shrub cover. Shrub cover generated non‐linear responses across all four bird diet groups and four of the five nesting groups. Species richness increased over time, with changes in the composition of bird assemblages being driven more by changes in shrub than tree cover. With recent predictions indicating a potential increase in shrub cover that is driven by global factors, community‐wide changes in bird communities may intensify. To minimize negative consequences of changes in bird communities, land managers should initiate and expand existing woody cover management regimes in southern African savannas, where shrub cover remains high.

## Graphical abstract

Global open terrestrial ecosystems continue to be impacted by shrub encroachment. We found that an increase in shrub cover reduced bird species occupancy and altered community assemblage in an African savanna. Land managers can minimize biodiversity impacts by controlling shrub density within conservation areas.

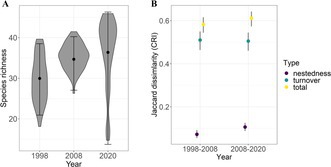

## 1 Introduction

Land‐cover change, driven by direct anthropogenic effects (e.g., habitat loss) and global climate change (Harnik et al. 2012), is one of the major drivers of biodiversity loss (Young et al. 2016). One rapidly increasing land‐cover change occurring in savannas and open terrestrial systems is an increase in woody cover from shrubs. Shrub encroachment, or the increase in the density of shrubs over time (Eldridge et al. 2011), is occurring across terrestrial biomes and contributes to ecosystem shifts from open to closed canopy systems (Parr et al. 2012). Broad‐scale changes such as increases in rainfall regimes, elimination of large herbivore communities, or changes in land use and management have been associated with shrub encroachment (Skowno et al. 2017; Stevens et al. 2016). Moreover, this phenomenon is often linked to increased atmospheric carbon from global climate change and is predicted to intensify in the coming decades (Eldridge et al. 2011; Stevens et al. 2016).

Shrub encroachment is particularly common in savanna ecosystems and its effects are predicted to intensify in the coming years in Africa (Stevens et al. 2017). Shrub encroachment can lead to savanna homogenization, reduced species richness, and shifts in faunal communities, with potential changes in ecosystem functions and services (Parr et al. 2012; Loggins et al. 2019; Sirami et al. 2009; Sirami and Monadjem 2012; Stanton et al. 2018; Wangai et al. 2016). Alternatively, an increase in shrub cover may benefit some vertebrate communities (Stanton et al. 2021) and may enhance some ecosystem services (Eldridge and Soliveres 2014).

Despite these potential implications, identifying generalizable trends in the direction and magnitude of shrub encroachment effects on vertebrate communities remains elusive for several reasons (Eldridge et al. 2011; Stanton et al. 2018). First, many studies examining effects of shrub encroachment use space‐for‐time substitution (e.g., Stanton et al. 2021; Sirami et al. 2009), thereby assuming species respond similarly over time as observed across space. However, the problem of shrub encroachment is inherently temporal and consideration of temporal changes in species composition and turnover may provide more reliable insights into shrub encroachment effects than space‐for‐time studies. In this context, changes over time can be driven by species substitutions (turnover or replacement) or changes in species richness (i.e., ‘species richness differences’), such as increasing nestedness (i.e., species subsets) in communities over time (Legendre 2014; Legendre and Gauthier 2014). Long‐term studies are needed for capturing such nuanced and important community variation in changing landscapes (Fukami 2004; Cadotte and Fukami 2005), yet long‐term studies on the effects of shrub encroachment are rare (but see Sirami and Monadjem 2012). Second, while our knowledge of the dynamics of shrub encroachment is advancing, increases in tree density are also common (Coetsee et al. 2023; Western and Maitumo 2004) and may obscure understanding the effects of shrub encroachment per se versus general changes in woody cover (e.g., Stevens et al. 2017). Because woody vegetation can promote habitat heterogeneity and therefore biodiversity (Tews et al. 2004), understanding various components of woody cover would facilitate conservation within open ecosystems such as savannas. Finally, understanding the extent to which species traits may help explain species responses to shrub encroachment is needed for identifying generalizable patterns, yet our understanding of the extent to which species traits help predict effects remains limited (but see, e.g., Stanton et al. 2021).

We investigated the relative effects of long‐term changes in shrub cover and tree cover on bird communities in an African savanna over a 22‐year period. We quantified changes in shrub, tree, and grass cover for 3 years over this time period (1998, 2008, and 2020) using satellite imagery. We then linked patterns of shrub or tree cover change with bird surveys to understand the dynamics of bird assemblages. We asked three key questions. First, how have vegetation cover and bird assemblages changed over 22 years? Second, are changes in shrub cover or tree cover more important in explaining changes in bird species occupancy and community composition? Third, do bird species traits explain changes in occupancy? We expected an increase in shrub cover based on prior reports in the region and other changes in woody cover across southern Africa (Stevens et al. 2017; Bailey et al. 2016; Roques et al. 2001). We predicted that the relationship between shrub cover and species richness would be hump‐shaped, as moderate levels of shrub cover can increase habitat availability for some species (McCleery et al. 2018; Sirami et al. 2009). Because changes in woody cover can alter the availability of nesting substrates and influence vegetation structure for foraging, we expected traits related to nesting and foraging strategies to explain variation in effects of woody dynamics. For instance, we expected a negative effect of shrub cover on species reliant on grasses for nesting and/or foraging, as shrub cover may reduce grass cover for grass‐nesting species and thereby reducing potential food for species that primarily forage on seeds. In contrast, an increase in tree cover could benefit cavity‐nesting species.

## 2 Methods

### 2.1 Study Area

We conducted this study in the Lowveld of Eswatini, which is characterized by a low‐lying savanna ecosystem. The Lowveld savanna ranges from 150 to 600 m in elevation and the savanna is characterized by a distinct dry (winter; May–September) and wet (summer; October–April) season. The summers are relatively warm (mean~26°C in January) and contain most of the annual rainfall, which ranges from 500 to 700 mm (Monadjem and Garcelon 2005; Monadjem and Reside 2008). Winters are cooler (mean~18°C in July) and dry (Monadjem and Garcelon 2005). The Lowveld is a critical component of the globally recognized high biodiversity system known as the Maputaland‐Pondoland Albany biodiversity hotspot (Perera et al. 2011). The ecosystem is threatened by expanding commercial agriculture and other human activities such as cattle grazing, which often lead to land degradation (Bailey et al. 2016), as cattle fail to control shrubs (Calleja et al. 2019) and overgrazing frequently leads to shrub encroachment through reductions in fuel load (O'Connor et al. 2020).

**FIGURE 1:**
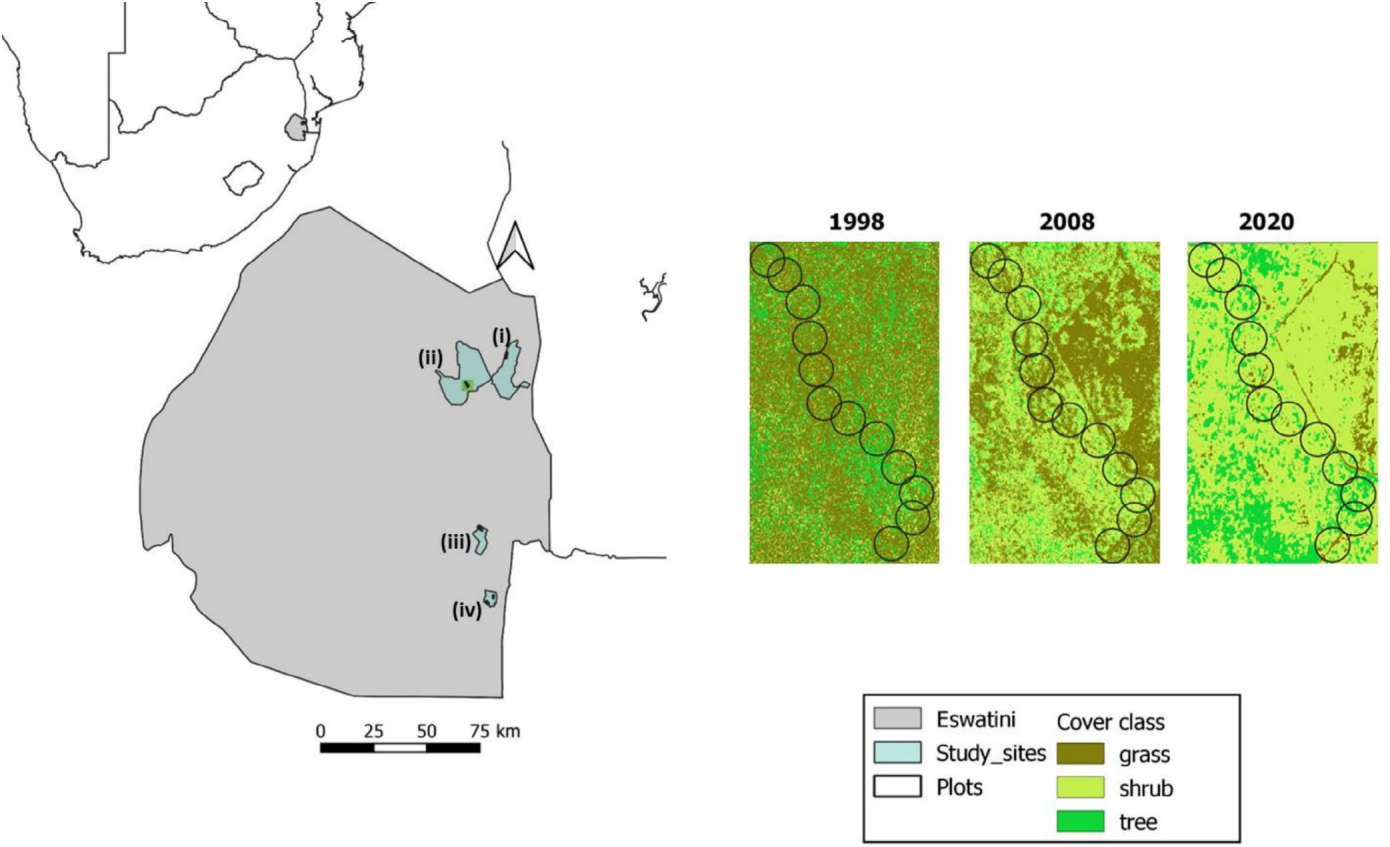
Map showing the study sites located in the eastern part of Eswatini. Also shown is the arrangement of 12 study plots in one of the sites and vegetation cover maps for 1998–2020. Mlawula, Hlane, Mhlosinga, and KaMsholo are shown by, i, ii, iii, and iv, respectively.

We conducted this study in four different protected savannas in the Lowveld that were originally surveyed by Monadjem (2005) and Sirami and Monadjem (2012): Mlawula Nature Reserve (Mlawula), Hlane Royal National Park (Hlane), Mhlosinga Nature Reserve (Mhlosinga) and KaMsholo Bushveld Safaris (formerly known as Nisela Safaris; Figure 1). Protected savannas in Eswatini are predominantly managed as proclaimed national or privately owned game reserves. For example, Mlawula and Hlane are proclaimed national reserves whereas Mhlosinga and KaMsholo are privately owned. Additionally, these sites vary in size, with Hlane (21,735.8 ha) and Mlawula (16,292.4 ha) being much larger than Mhlosinga (3742 ha) and KaMsholo (1147 ha). These sites are dominated by trees such as *Senegalia (Acacia) nigrescens*, *S. tortilis* , *Sclerocarya birrea caffra*, and a native shrub that has encroached on savannas across the region, the sicklebush *Dichrostachys cinerea* (Roques et al. 2001). Thickets of sicklebush can create dense shade and hence reduce ground cover (Randle et al. 2018). These sites lack elephants, which are an important woody cover disturbance agent and can alter shrub cover dynamics, potentially interacting with other types of herbivory (e.g., grazing), fire and drought regimes (Staver et al. 2009; Roques et al. 2001; Sankaran et al. 2005). Between 2008 and 2020, KaMsholo management cleared savanna vegetation across a portion of our study area to plant sugarcane. Based on satellite images, land clearing commenced around 2009, and sugarcane was planted between 2010 and 2011. Sugarcane farming continued up to 2017, after which the area remained uncultivated with natural vegetation starting to grow back. By 2020, it was dominated by a thick grass layer and a few shrubs including some remnants of sugarcane. We address this change at KaMsholo below.

### 2.2 Bird Counts

A total of 48 plots (12 plots in each of the four sites) were established in 1998 (Monadjem 2005). Each plot was at least 200 m away from its nearest neighbor to reduce double counting of individuals across plots. Plots were placed at least 200 m away from riparian zones since riparian areas largely encompass different bird communities (Monadjem 2005). Each year, one or two experienced observers conducted point counts (Thompson 2002; Monadjem 2002) during the breeding or wet season, that is, October to March (1998: AM; 2008: AM and CS; 2020: MS). We conducted 10‐min counts and recorded all birds that we saw or heard within a 100 m radius of the center of a plot, therefore obtaining data on species abundance within each point. The choice of 100 m radius has been shown to maximize species detection per unit area surveyed, leading to an accurate assessment of bird community structure (Ralph et al. 1995). We excluded species belonging to groups such as waterbirds and raptors as these are not amenable to the point‐count technique and are not expected to respond to attributes of plot vegetation cover. We restricted counts between dawn and four hours after dawn because bird activity is highest. We conducted a total of four surveys per plot and year while randomizing the starting time in each plot to reduce potential time‐of‐day effects. All bird counts were conducted in the wet season, the peak breeding season, such that each plot was surveyed once per month from November (1998) to February (1999) during the 1998 wet season whilst surveys for 2008 and 2020 were completed within December. Despite these different time ranges, there is evidence that variation in the bird community in December captures seasonal patterns in our study area (Sirami and Monadjem 2012). We surveyed plots on consecutive days in 2008 and 2020. We did not count birds when it was raining or when the wind speed exceeded 20 km/h as these conditions could reduce bird activity or impair detection. The four counts per season allow for increasing the chances of detecting a species when present and therefore minimizing false absences (MacKenzie et al. 2002).

### 2.3 Vegetation Cover

We quantified three attributes of the savanna vegetation (tree, shrub, and grass cover) to assess overall habitat change between 1998, 2008, and 2020. We used black and white aerial photographs (orthophotos) at 1 m resolution, SPOT5 satellite images at 2.5 m, and Planet scope satellite images at 3 m resolution for 1998, 2008, and 2020 respectively. The selection of satellite data was guided by the availability of high‐resolution data for the region for each period. While the freely available Landsat imagery data spans the 22 years, its relatively coarse resolution is not sufficient to assess shrub encroachment, which requires high‐resolution imagery. Since the orthophotos contained a single band, we conducted a texture analysis to enhance pixel differentiation, using the Gray Level Co‐occurrence matrix (Haralick et al. 1973) in the Orfeo ToolBox (OTB) in QGIS 3.12.0 (QGIS Development Team 2020). Specifically, we used the simple Haralick texture feature in OTB. The simple Haralick feature has eight local features that are likely correlated; therefore, to minimize correlation between features, we used Principal Components Analysis (PCA) to select three features that explained most of the spectral variation. In contrast, as the SPOT5 and Planetscope images exhibited color bands that enhanced visualization, we did not conduct texture analysis.

We predicted the occurrence of trees, grass, and shrubs using the random forest (RF) classifier in the Google Earth Engine (GEE) platform (Gorelick et al. 2017). We trained the classifier by providing user‐defined points based on polygons that were constructed from similar pixels using our knowledge of the study system. For example, the riparian vegetation is dominated by trees near waterways in our system; we therefore used these pixels to select tree training points. We assessed grass cover by identifying patches with open canopy such as some plots in KaMsholo (see Study area section above), areas whose woody vegetation was cleared during land management practices, and other open savanna patches. We incorporated bare ground (mostly visible from dirt roads) into our grass cover class because initial exploration suggested that the RF classifier could not effectively separate the two cover types. In this context, our grass cover category describes open savanna patches without woody vegetation as observable from above and not the quantity of grass under trees and shrubs. We assessed shrub cover by treating all pixels that did not include grass or tree cover as potential shrub‐cover pixels. On average, each cover class was represented by 39 randomly selected polygons whose distribution was proportional to the size of our four study sites. We randomly assigned points for model training (70%) and validation (30%). We assessed the accuracy of our classification using the confusion matrix and associated statistics (Congalton 1991): the Kappa statistic, overall accuracy, producer and user accuracy (*sensu*, Congalton and Green 2008).

### 2.4 Bird Traits

To understand the effect of species traits on species response to woody cover changes, we used Roberts Birds of Southern Africa 7th Edition (Hockey et al. 2005), which is a well‐developed and globally recognized bird species' life history traits literature for southern Africa. We classified birds based on habitat, diet, and nesting traits. First, we classified species according to their preference for three habitats: grassland, forest, and savanna woodland. We used these categories as a coarse classification for interpreting whether the community was dominated by more ‘open’ (grassland) species or more ‘closed’ (forest) species, as such species should respond very differently to changes in woody cover dynamics (Péron and Altwegg 2015; White et al. 2024). Species‐habitat associations were classified using integers from 0 to 3 in Hockey et al. 2005, describing no association (0), occasional (1), secondary (2) and primary (3) association with habitat. We classified species as grassland and forest specialists when grassland and forest ecosystems formed their primary habitat, and as savanna woodland specialists otherwise. For each habitat category, we calculated the number of species detected during each year. Regarding the diet category, many species consume more than one food type, so we classified species according to their dominant diet preferences, as suggested by Hockey et al. (2005). For instance, since granivores (seedeaters) increasingly consume arthropods during the breeding season (i.e., when we conducted our surveys), we classified them as granivores to capture their dominant diet attributes. Finally, we classified species based on their nesting substrate preferences: tree, shrub, cavity, or grass. Three species from our bird community are brood parasites (diederik cuckoo *Chrysococcyx caprius* , klaas's cuckoo *Chrysococcyx klaas* , red‐chested cuckoo *Cuculus solitarius* ) and were classified as ‘parasites’ in our analysis.

### 2.5 Analysis

#### 2.5.1 Trends in Vegetation Cover

To quantify trends in vegetation cover where birds were sampled, we calculated the proportion of grass, shrub and tree cover per plot for each time period. To test for changes in vegetation cover over time, we employed generalized linear mixed models (GLMMs) within the *glmmTMB* package (Brooks et al. 2017) and incorporated year as a categorical variable to test for long‐term trends and plot as a random effect. Since the distribution of all three vegetation cover components did not meet assumptions of normality, we modeled all proportion (0–1) data with a beta distribution. We also added a 0.05 constant to measures of shrub and tree cover to prevent models from being undefined when the cover was zero. We tested for pairwise comparisons between years using *pairs* and the *emmeans* function within the *emmeans* package (Lenth 2024). A total of five plots were cleared of all woody vegetation between 2008 and 2020 in KaMsholo, so we also tested for change in cover with and without these plots. The inclusion of these plots did not significantly alter results on grass, shrub, and tree cover trends (Figure S1); and we therefore included these plots in all subsequent analyses to keep the same spatial locations previously sampled by Monadjem (2005) and Sirami and Monadjem (2012). Finally, we investigated changes in each vegetation class (grass, shrub, and tree) per site to understand whether changes were consistent across sites over time. To do this, we fitted year by site interaction models, where we treated ‘year’ as a categorical variable and used *pairs* and the *emmeans* function from the *emmeans* package to compare site‐specific variability in vegetation cover between years.

#### 2.5.2 Occupancy Modeling

We fitted a hierarchical multispecies occupancy model that accounts for species‐specific occupancy and detectability (Dorazio et al. 2010; Royle and Dorazio 2009). The four surveys (visits) per plot per season, allowed for estimating species detectability. This model parameterization allows community‐wide inferences by including hyperpriors and hyperparameters that capture parameter variation across the entire community. The strength of multispecies occupancy models lies in tracking species‐specific effects while sharing information that allows effective community‐wide inference (Iknayan et al. 2014). Since multispecies occupancy models may be less well suited for the rarest species within the community (Royle and Dorazio 2009), we compared average occupancy effects and trends in species richness from a model based on all species to a model based on species recorded in more than 10% of our plots. Both trends in species richness (Figure S2) and effects at the community level (Figure S3) from the model incorporating all species were qualitatively similar to the data subset. We here present results for a model based on species that were recorded in at least 10% of the plots (see Table S1 for species and the associated traits).

We fitted a model that allowed for non‐linear effects of shrub and tree cover on occupancy by including a quadratic term, which was allowed to vary across species (see model statement below). We accommodated for potential curvilinear effects of shrub and tree cover using quadratic terms because some studies have shown non‐linear responses of diversity to woody cover (McCleery et al. 2018; Sirami et al. 2009). We did not include grass cover in this model, because it was highly correlated with shrub cover (*r* = −0.8). Our model parameterization included species‐specific random effects of year on occupancy to account for unmeasured potential factors such as droughts (Tfwala et al. 2020). We allowed for detection probability to vary across species by considering species as random intercepts. We generated posterior distributions of our parameters using the *jagsUI* package (Plummer 2003; Kellner 2016) in R version 4.0.3 (R Core Team 2020). In this model, we specified vague priors and hyperpriors (Gelman et al. 1995) for our model parameters, with covariates having normally distributed priors with a mean of 0 and precision of 0.6 (Northrup and Gerber 2018). We ran four chains of 20,000 iterations, a burn‐in of 4000 iterations, and at a thinning rate of 20, yielding a total of 3200 posterior samples. We visualized the trace plots and the Gelman‐Rubin diagnostics, whereby observations of values ≤ 1.1 suggested model convergence (Gelman and Rubin 1992; Gelman and Hill 2007). We specified the model as follows:

$$\text{logit}\left(\psi_{\mathrm{itk}}\right)=\beta 0_{k}+\;\beta 1_{k}{\text{shrub}}_{\mathrm{it}}+\beta 2_{k}{\text{shrub}}_{\mathrm{it}}^{2}+\beta 3_{k}{\text{tree}}_{\mathrm{it}}+\beta 4_{k}{\text{tree}}_{\mathrm{it}}^{2}+\omega_{k}$$

$$\text{logit}\left(\rho_{\text{ijkt}}\right)=\alpha 0_{k}$$

where *ψ*_itk_ describes the probability of occupancy of species *k* during year *t* at plot *i*. The coefficients, β1 and β2 explain variation in occupancy due to linear and non‐linear effects of shrub cover whereas β3 and β4 explain linear and non‐linear effects of tree cover, respectively, whereas 𝜔 denotes year as a random variable to control for year effect. On the other hand, *ρ* describes species‐specific detection probability during survey (sampling period) *j*. The coefficients, β0 and denotes species random intercepts for occupancy while α0 describes species random intercepts for detectability. Since bird counts were conducted in the first 4 h after dawn by the three observers in various years (1998: AM; 2008: AM and CS; 2020: MS), we tested a model that allowed species detectability to vary by time and observer and found that it was quantitatively similar to a simpler model that only treated species detectability as a random intercept as shown by our model statement.

We considered responses to be ‘significant’ if the 95% credible intervals (CRI) of parameters did not overlap zero, but also considered a less stringent criterion, that is, 90% CRI. We considered a significant response to be positive or negative if occupancy varied significantly with only the linear term, and curvilinear if the quadratic term was significant. For species with significant curvilinear relationships, we estimated the cover of shrubs or trees at which occupancy peaked by calculating the first derivative concerning shrub or tree cover and solving the resulting function when set to zero (i.e., identifying where the slope is zero along the non‐linear gradient; Ewers and Didham 2006). We evaluated variation in species richness by summing the estimated latent occupancy state (0, 1) for each species based on the four surveys per plot each year. Furthermore, we quantified plot‐level mean community dissimilarities between years to interpret temporal beta diversity (species turnover in plots over time). To accomplish this, we used the 3200 posterior samples of the site by species matrix and summarized measures of community composition at the plot level and across years using the *betapart* package in R (Baselga and Orme 2012). We computed the Jaccard index, which was then partitioned into turnover and richness differences in plots between years (Baselga 2012). To test whether species richness varied over time, we ran a regression using the *lme4* R package (Bates et al. 2015) with site as a random effect and year as a fixed effect. Finally, we used correlation to evaluate the strength and direction of the influence of vegetation cover dynamics on changes in species richness.

## 3 Results

### 3.1 Changes in Vegetation Cover

**FIGURE 2:**
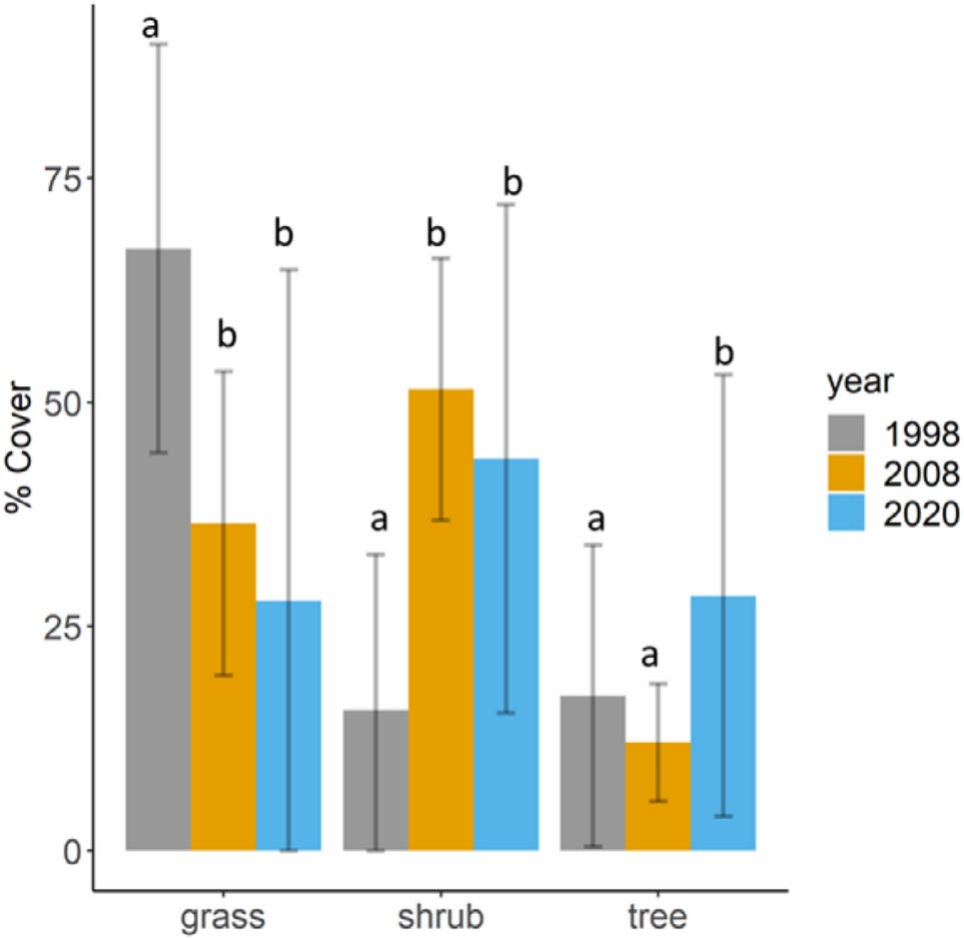
Overall change in the three vegetation cover types: grass, shrub and tree in the low‐lying savanna of Eswatini as quantified in the wet season between 1998, 2008 and 2020. The bar graphs indicate year‐specific average cover and also shown are the standard errors (bars) for each cover type during each period. Letters, ‘a’, ‘b’ and ‘c’, represent pairwise comparisons of measures with different letters suggesting a significant difference while shared letters indicate non‐significance.

**FIGURE A1:**
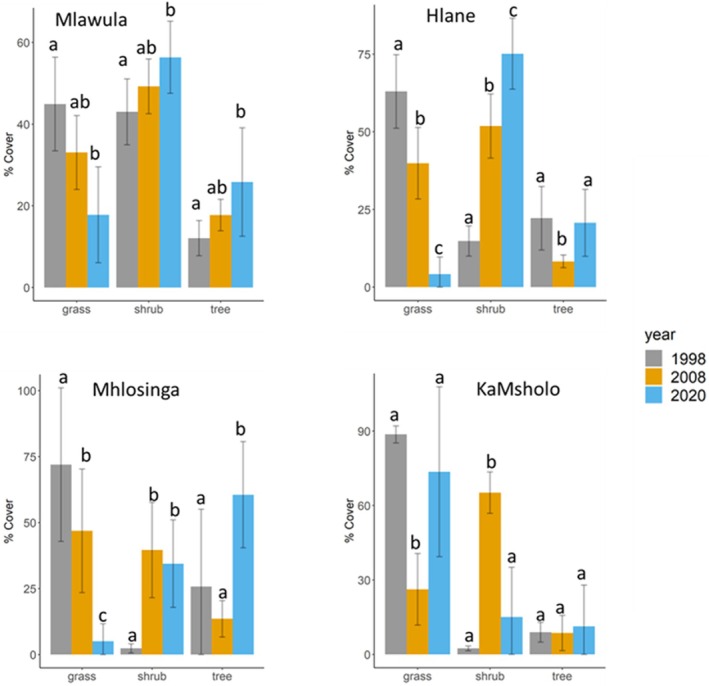
Vegetation cover (grass, shrub, and tree) dynamics in four protected areas across the low‐lying savanna ecosystem of Eswatini assessed between 1998, 2008 and 2020 during the wet season. Letters (a, b, and c) represent pairwise comparison results of class variation between years in each site with same letters suggesting no difference and dissimilar letters suggesting a significant difference in cover.

Our RF classifier of vegetation cover was highly accurate (Kappa > 0.95 each year; Table S2). Overall, grass cover decreased significantly by 39% between 1998 and 2020 (*β* = −0.66 ± 0.12, *p* < 0.001), whereas there was a 28% increase in shrub (*β* = 0.51 ± 0.10, *p* < 0.001) and an 11% increase in tree cover (*β* = 0.20 ± 0.08, *p* = 0.002). When testing differences between years, we found that grass and shrub cover significantly decreased and increased, respectively, only between 1998 and 2008 (Estimate grass = −0.86, SE = 0.24, *p* = 0.001; Estimate shrub = 1.42, SE = 0.19, *p* < 0.0001) and 1998–2020 (Estimate grass = −1.33, SE = 0.24, *p* < 0.0001; Estimate shrub = 1.09, SE = 0.19, *p* < 0.0001), but did not vary between 2008 and 2020 (Estimate grass =0.47, SE = 0.24, *p* = 0.115; Estimate shrub = −0.33, SE = 0.18, *p* = 0.144; Figure 2). On the other hand, tree cover significantly increased both between 1998 and 2020 (Estimate = 0.39, SE = 0.17, *p* = 0.05) and 2008–2020 (Estimate = 0.57, SE = 0.17, *p* = 0.002) and not between 1998 and 2008 (Estimate = −0.18, SE = 0.18, *p* = 0.548; Figure 2). Essentially, change in vegetation cover varied across sites: Hlane and Mlawula (proclaimed national reserves) tended to exhibit consistent increasing trends in shrub cover, whereas Mhlosinga and KaMsholo (private reserves) exhibited declines in shrub cover after 2008 (Figure A1).

### 3.2 Effects of Vegetation Cover on Species‐Specific and Average Species Occupancy

**FIGURE A2:**
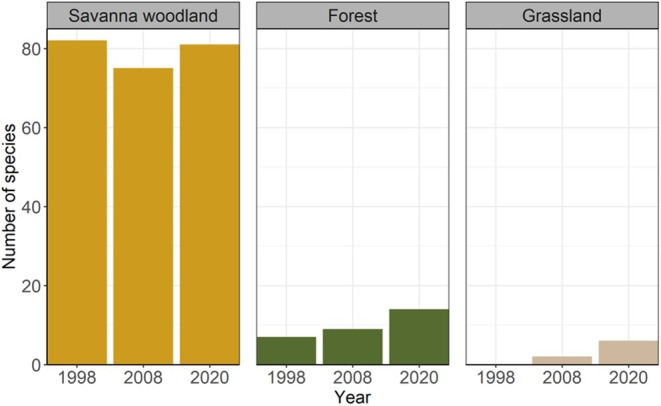
Habitat associations for the 121 bird species assessed in the low‐lying savanna ecosystem of Eswatini during the wet seasons of 1998, 2008 and 2020. Shown is the number of species that generally prefer open grasslands, forests, and savanna woodlands (close canopy savannas).

We recorded a total of 121 bird species across the years, with 97, 90, and 110 species detected in 1998, 2008, and 2020, respectively. The 121 species were dominated by savanna woodland species (51%). Forest‐adapted species increased from 7 to 14 species between 1998 and 2020 (Figure A2). Grassland species only increased on the five plots that were cleared of woody vegetation between 2008 and 2020, such that out of the total of six species, four (African pipit *Anthus cinnamomeus* , croaking cisticola *Cisticola natalensis* , fan‐tailed widowbird *Euplectes axillaris* , and zitting cisticola *Cisticola juncidis* ) were only detected on these five plots. Out of the total of 121 species, 64 were detected in ≥ 10% of the plots and therefore formed the core of our analysis. Of these 64 species, 62, 61, and 63 were encountered in 1998, 2008, and 2020, respectively.

**FIGURE 3:**
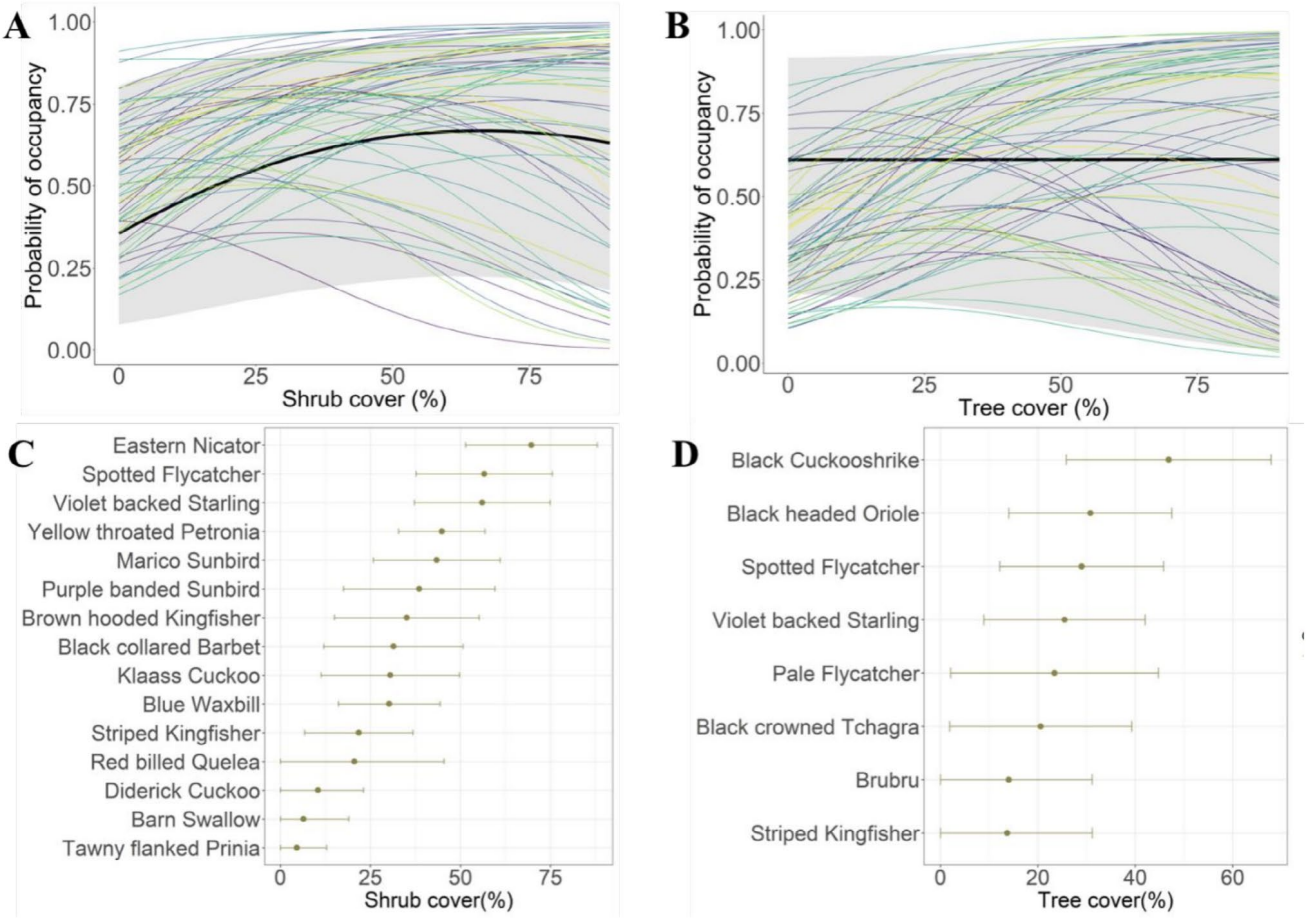
Community mean (thick black line) and species‐specific occupancy (thin lines) responses to shrub (A) and tree (B) cover for bird species assessed in the low‐lying savanna ecosystem of Eswatini during the wet seasons of 1998, 2008 and 2020. The gray ribbon delineates the 95% CRI for community responses. Specifically, species exhibited peak occurrence responses at various levels of shrub (C) and tree (D) cover. Shown are means (dots) and standards errors (error bars) for only species that exhibited significant curvilinear responses at 95% CRI.

**FIGURE 4:**
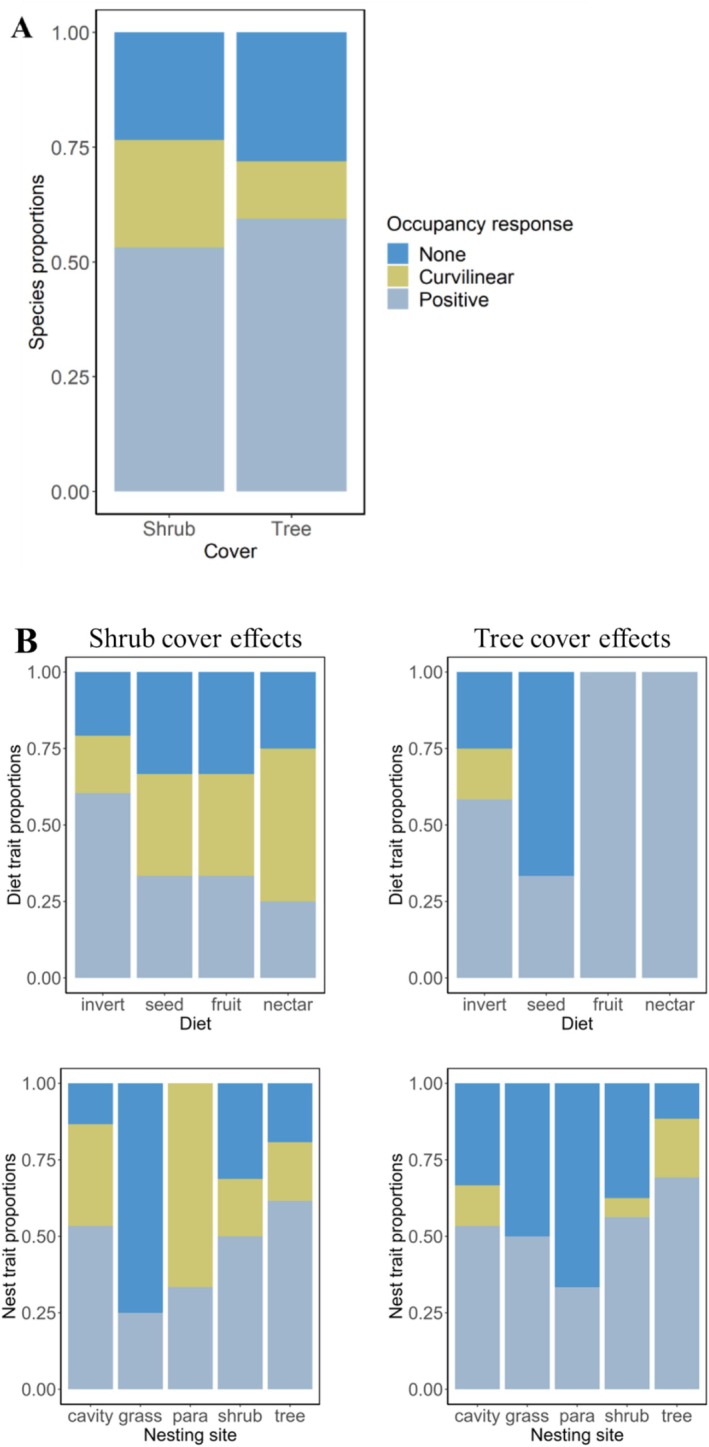
Species occupancy response (A) and diet and nesting trait (B) effects of shrubs and trees on bird communities assessed in the low‐lying savanna ecosystem of Eswatini during the wet seasons of 1998, 2008 and 2020. Species proportions are based on the 64 species assessed in our model and their responses to shrubs or trees. Significant responses were determined if their 95% CRI did not overlap zero. Diet and nesting trait responses were determined through posterior summaries of *β*s for shrub and tree cover to characterize responses of species sharing similar traits. See Table S1 for species trait information.

We found evidence for a community‐wide effect of shrub cover (Figure 3A), but not tree cover (Figure 3B), on average species occupancy. Average species occupancy increased non‐linearly with shrub cover (*β* = 3.92, CRI = 3.08, 4.80; *β* ^2^ = −3.00, CRI = −4.17, −1.76). Based on the 95% CRIs of estimated coefficients, 34 (53%) species exhibited positive linear associations with shrub cover, whereas 15 (23.4%) had significant curvilinear responses to shrub cover (Figure 4A), with occupancy patterns generally peaking at < 50% cover (Figure 3C). However, when considering the 90% CRIs, the positive response to shrub cover dropped to 40.6% while the curvilinear response increased to 57.8%. Regarding associations with tree cover, 38 (59%) species exhibited positive linear associations, while eight (12.5%) exhibited curvilinear responses (Figure 4A); with curvilinear respondents peaking at < 40% cover (Figure 3D). At 90% CRIs, positive linear responses to tree cover dropped to 36 species while curvilinear responses increased to 28 (43.8%) species. Based on the 95% CRIs, variation in shrub and tree cover could not explain the occupancy of 15 (23%) and 18 (28%) species, respectively. Across diet and nesting traits, we found consistent curvilinear effects of shrub cover; frugivores, nectarivores, and grass‐nesting species exhibited a positive association with tree cover (Figure 4B).

### 3.3 Effects of Vegetation Changes on Species Richness and Community Composition

**FIGURE 5:**
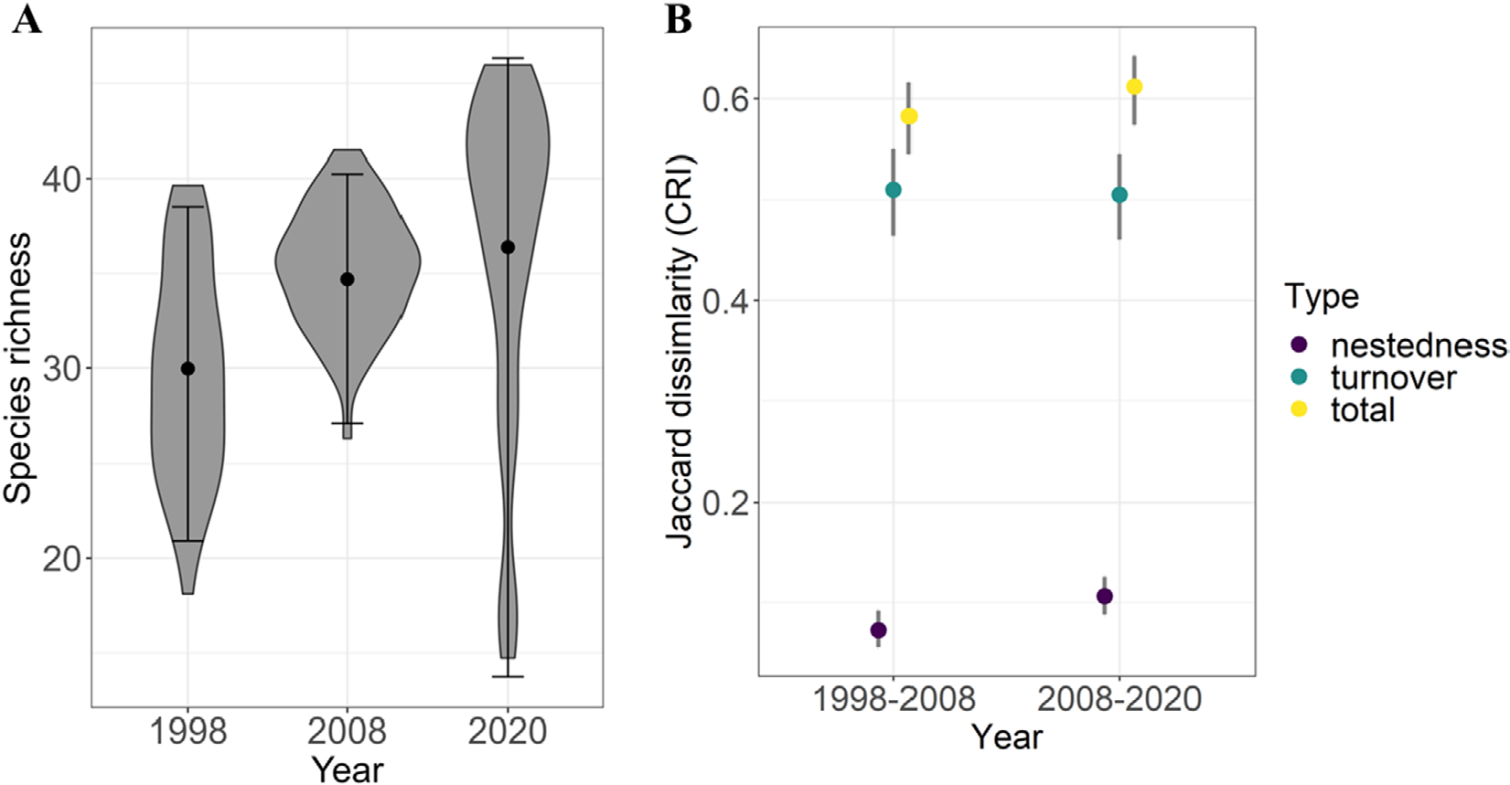
Estimated species richness (points = mean, error bars 95% CRI), with violin plots showing posterior distributions (A) and temporal beta diversity measures (B) for communities assessed in the low‐lying savanna ecosystem of Eswatini during the wet seasons of 1998, 2008 and 2020.

Based on our model that incorporated a total of 64 species, the estimated mean species richness per plot changed from 30 to 36 between 1998 and 2020 (Figure 5A), indicating an upward trend in the number of species occurring in our plots over the 22 years (*β* = 3.2, 95% CI = 2.1, 4.3, *p* < 0.001). Species richness varied more widely among plots in 2020 than in other years, with some plots having < 20 species and others > 40 species (Figure 5A) and this pattern remained the same when we excluded the plots that were cleared in KaMsholo between 2008 and 2020. The Jaccard dissimilarity index (total) quantified among pairs of years did not change across periods, with a value of 0.58 for 1998–2008 and 0.61 for 2008–2020 (Figure 5B). This community dissimilarity over time appears to be driven by species turnover rather than richness differences, with a higher contribution of turnover to dissimilarities (around 0.51 in each period) than richness differences (0.07 for 1998–2008 and 0.11 for 2008–2020; Figure 5B).

**FIGURE 6:**
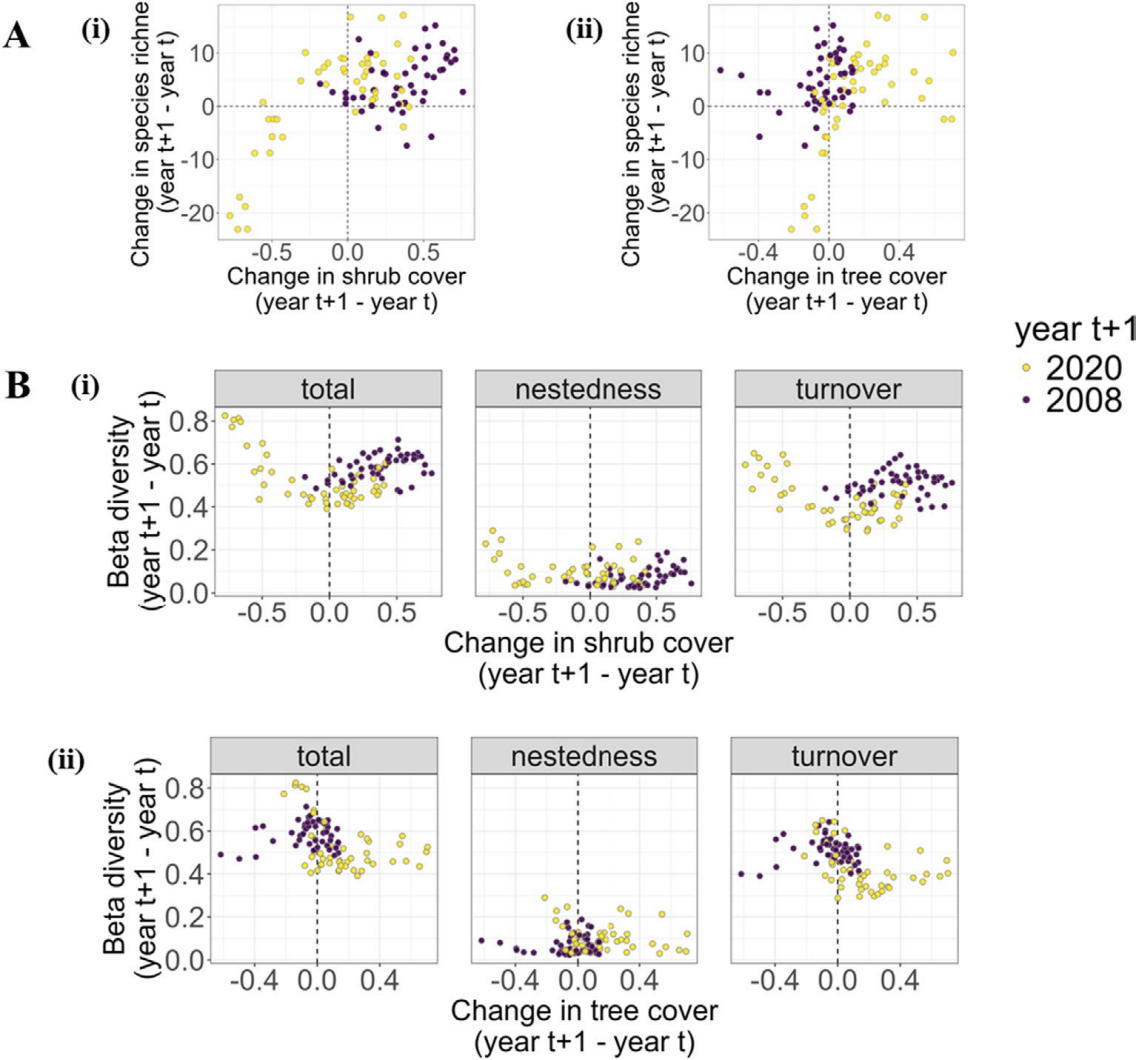
Effects of changes in shrub and tree cover on species richness (A, i and A,ii, respectively) and on dynamics of beta diversity (B,i and B,ii, respectively) of bird communities assessed in the low‐lying savanna ecosystem of Eswatini during the wet seasons of 1998, 2008 and 2020. From left to right in the B panel, each figure shows total beta diversity, richness differences and turnover rate. The dots indicate study plots and colors: Purple = 2008 (changes between 1998 and 2008) and yellow = 2020 (changes between 2008 and 2020).

The change in shrub cover between 2008 and 2020 was strongly correlated with changes in bird species richness (*r* = 0.72) (Figure 6A,i), while changes in tree cover during the same period were moderately correlated with species richness (*r* = 0.53) (Figure 6A,ii). Species richness was less correlated with both shrub‐ (*r* = 0.35) and tree‐cover (*r* = 0.25) dynamics between 1998 and 2008. Shrub cover impacts on the dynamics of species richness became stronger over time (steeper changes in richness between 2008 and 2020 than between 1998 and 2008). Shrub cover appeared to have greater effects on changes in community dissimilarity over time compared to tree cover (Figure 6B). These changes in shrub cover drove increasing trends of turnover and richness differences from 0.39 to 0.65 and 0.02 to 0.29 between 1998–2008 and 2008–2020, respectively, whereas the change in tree cover was not associated with temporal changes in beta diversity (Figure 6B,ii).

## 4 Discussion

Our results emphasize that shrub encroachment has widespread and ongoing effects on bird communities that are independent of changes in tree cover. Specifically, we found that changes in bird community composition are primarily due to species turnover. We discuss implications for biodiversity conservation in southern African savannas.

### 4.1 Shrub Encroachment Over 22 Years

We found a general increase in shrub cover over a 22‐year period using remote sensing. Over this period, shrub cover increased substantially in the first 10 years (%mean = 15.6, SD = 17.4 in 1998) and then largely stabilized between 2008 (%mean = 51.5, SD = 14.6) and 2020 (%mean = 43.7, SD = 28.4), a result likely driven by two main factors. First, the region experienced El‐Nino‐related droughts around the 2015/2016 season, which can increase mortality rates of woody vegetation (Fensham et al. 2009; Twidwell et al. 2014; Jones et al. 2022), with potentially stronger impacts on encroaching shrubs (Case et al. 2019). Second, land managers have recently adopted more intensive measures to control shrub encroachment to enhance benefits for both wildlife and tourists (Gray and Bond 2013). Across the four sites, we found that on average shrub encroachment in our plots consistently increased in Hlane and Mlawula (proclaimed national reserves) over the 22 years (Figure A1). However, Mhlosinga and KaMsholo (private reserves) showed inconsistent shrub cover dynamics. Private reserves practice regimented management of wildlife populations such as large herbivores and vegetation using fire and other means. Long‐term fluctuation in shrub encroachment can be driven by local variation in herbivore abundances, climate variability, and land management (Roques et al. 2001). Ungulate populations may impact vegetation cover: while browser activity may have a suppressive effect on shrubs (Augustine and Mcnaughton 2004), heavy grazing has been associated with shrub competitive release (O'Connor et al. 2014) and therefore the balance between browsing and grazing herbivorous groups is essential. Land management activities that involve clearing woody vegetation (through controlled burning or mechanical removal) may be prevalent within the private reserves in this study. According to Swaziland's Third National Communication to the United Nations Framework Convention on Climate Change (2016), precipitation has tended to decrease around the southern region within the savanna ecosystem of Eswatini (Eswatini Meteorological Service 2019), such that both Mhlosinga and KaMsholo (located in the south) could have experienced more severe effects from droughts such as the El‐Nino during 2015/2016. Indeed drought frequency and severity are increasing within Eswatini (Tfwala et al. 2020). On the other hand, average tree cover was highest in 2020 (mean = 28.4, SD = 24.6) and lowest in 2008 (mean = 12.0, SD = 6.5), suggesting the potential for trees to form a crucial vegetation component for wildlife within our system. Our results also indicate a strong negative correlation between shrub and grass cover (*r* = −0.8), suggesting that shrub encroachment may be reducing open, grassy savanna patches (Sirami and Monadjem 2012). Conversely, the lack of correlation between tree and shrub cover (*r* = −0.03) warrants a joint assessment of these savanna components to isolate their relative effects on faunal communities. Altogether, these results highlight the need for continuous assessment of potential wildlife impacts of woody cover dynamics within mesic savannas of southern Africa.

### 4.2 Shrub Encroachment Effects on Species Occupancy

Across all periods (1998, 2008, or 2020), the initial (1998) and subsequent (2008 and 2020) bird communities were dominated by woody‐adapted species (Figure A2), and we found that shrub cover dynamics tended to drive changes in species occurrence across the community. These results provide an important baseline for assessing the long‐term impacts of shrub encroachment in woody savanna ecosystems for two reasons. First, our results highlight the importance of considering species‐habitat associations by determining vegetation characteristics preferred by species when interpreting the effects of shrub cover (White et al. 2024). Second, our results decoupled shrub versus tree effects, an important step to fully understand the effects of increasing woody vegetation density across mesic savannas.

The role of shrub encroachment in altering faunal communities is increasingly considered (Sirami et al. 2009; McCleery et al. 2018), because species persistence depends on the overall tolerance of species to habitat change (Péron and Altwegg 2015). Our results on the absence (or very low and localized abundance) of grassland species in our study area (Figure A2, Grassland) support the hypothesis that open‐adapted birds have been displaced due to shrub‐encroached savannas (e.g., Péron and Altwegg 2015). Fifty years prior to this study, shrub density increased by ~29% across our system (Roques et al. 2001), such that open‐adapted species likely disappeared prior to our earliest surveys in 1998. The remaining communities are mostly woody‐adapted species and tend to show positive responses to the increase in shrub cover. Yet, our results suggest that high levels of shrub encroachment may have a negative impact even on these species. There are at least three possible reasons for the decline of woody‐adapted species at high levels of shrub cover. First, higher levels of shrub cover may restrict foraging and reproductive success (Wilcove 1985). Second, increasing shrub cover is likely to inhibit crucial savanna structural components, such as grass and herbs (Randle et al. 2018), thereby reducing key resources for several bird groups (Blaum et al. 2009; Koch et al. 2018). Finally, high shrub cover may increase predation risk (Lima 1993; Loggins et al. 2019; Lima and Valone 1991), leading to an overall avoidance of densely wooded savannas.

Our results suggest that increasing tree cover in our system had no strong community‐level impacts on species occupancy. Increasing tree cover was positively associated with the occurrence of some species, with a few species declining at higher levels of tree cover. This weak association may be due to the fact that increasing tree cover only impacts bird communities above a certain threshold in terms of tree density, tree size and quality. Nonetheless, trees may enhance species richness by stimulating local colonization in shrub‐encroached savannas (Sirami and Monadjem 2012). Large trees can augment habitat heterogeneity by providing a suite of ecological benefits (Lindenmayer 2017), including nesting sites, refugia from predators, and diverse foraging sites.

Consideration of species‐specific or group responses, such as species traits, can provide key insights when assessing the impacts of shrub encroachment on vertebrate communities (Andersen and Steidl 2019). Contrary to our predictions, grassland species, such as most granivores (seedeaters), did not exhibit a negative association with shrub cover. The occurrence of some granivore species was positively, albeit marginally associated with shrub cover, such as the white‐winged widowbird *Euplectes albonotatus* whose habitat is shrinking at the regional scale (Péron and Altwegg 2015). Generally, most granivorous species are gregarious and highly mobile with the potential to effectively navigate remnant open patches within the system. However, grassland species that are granivores are strongly influenced by perceived predation risk (Verdolin 2006), and increasing shrub density may act as a proxy for risky patches and drive widespread habitat avoidance (Loggins et al. 2019), forcing some species such as red‐billed quelea *Quelea quelea*, which showed a strong curvilinear association with shrub cover, to find alternative refuge in surrounding human‐modified landscapes (Péron and Altwegg 2015; Lukhele et al. 2021). Meso‐carnivore communities are also changing with woody cover, and increasing chances of nest predation (Blaum et al. 2007; Crowley et al. 2025). Essentially, grassland species may readily exploit previously shrub‐encroached savanna patches that are dominated by grass cover, such as the four out of a total of six grassland species that occurred only across the five plots that were cleared of woody vegetation between 2008 and 2020 in KaMsholo. These five plots, however, were void of most savanna woodland bird species, potentially suggesting that to maximize species diversity, there is a need for maintaining some woody cover. On the other hand, the increasing occurrence of woody thicket‐adapted species such as the green‐backed camaroptera *Camaroptera brachyura* , southern boubou *Laniarius ferrugineus* and eastern nicator *Nicator gularis* suggests a potential shift in community composition and associated ecosystem services.

### 4.3 Impacts of Woody Cover Dynamics on Bird Community Change

Shrub encroachment can alter floral and faunal communities across various open ecosystems (de Souza et al. 2022; Furtado et al. 2021; Sirami et al. 2009; Andersen and Steidl 2019). Even though most of these patterns are associated with space‐for‐time studies (e.g., Stanton et al. 2021), our results suggest similar woody‐mediated variations in bird communities over time. We showed that long‐term community changes at a local scale are largely driven by species substitutions or turnover. Furthermore, because richness differences tended to increase over the 22 years, it highlighted that the overall gain of species over time was not consistent across all plots or sites. Our results also point out the importance of long‐term approaches to understanding the impacts of shrub encroachment on biodiversity and that when shrub cover remains high, beta diversity components may explain variation in community structure at various periods.

While projecting impacts of shrub encroachment on community change is critical (e.g., White et al. 2024), especially across Africa (Stanton et al. 2018), determination of base communities is challenging and therefore effects of shrub encroachment should be interpreted with caution, especially for sensitive groups such as birds associated with relatively open savanna grasslands that have largely disappeared across most shrub‐encroached savannas. However, this also suggests that conservation efforts should prioritize increasing open savanna grasslands by minimizing shrub cover and monitoring bird responses. While shrubs can sometimes outcompete grasses, ungulates may also impact grass production (Staver et al. 2021) and hinder the availability of critical resources to most savanna birds. In our system, private reserves, which are relatively smaller, adopted strong ungulate population control measures while national parks are marked by inconsistent efforts and therefore likely suffer from overgrazing. On the other hand, large trees augment habitat heterogeneity by providing a suite of ecological benefits (Lindenmayer 2017) including nesting sites, refugia from predators and a wider range of foraging opportunities. This keystone savanna structure is threatened by human activity in the region (Bailey et al. 2016). In our study sites, trees are under limited threat from local communities; however their distribution is threatened by climate change (Mtsetfwa et al. 2023).

## 5 Conservation Implications

We showed that the effect of shrub encroachment on bird assemblages over a 22‐year period led to high rates of species turnover and an increasing number of woody adapted species, supporting predictions about shrub encroachment driving grassland species population declines (White et al. 2024) and displacement by closed‐canopy species at the regional scale (Péron and Altwegg 2015). However, our species trait analysis suggests that shrub encroachment has the capacity to affect all diet and most nesting groups in our system. Altering bird diet groups may disrupt ecosystem services and lead to weakened and vulnerable ecosystems (Whelan et al. 2008). Given that many species showed non‐linear relationships with shrub cover, declining in occurrence at high levels, we recommend management of shrubs such as sickle bush *Dichrostachys cinerea* in the system by focusing on the most encroached areas.

We showed that shrub encroachment was consistent across two national parks but exhibited inconsistent changes within privately‐owned parks between 2008 and 2020. Both private reserves occupied the driest part of the savanna ecosystem (Tfwala et al. 2020), which may have influenced vegetation dynamics (e.g., Jones et al. 2022). Additionally, both the management of vegetation cover and large herbivore populations may have influenced the observed shrub cover dynamics. To maintain high‐quality savannas, national reserve management could prioritize leveraging available human and financial resources such as working with local communities who need wood resources for fuel and other uses instead of hiring employees to manage shrubs. Currently, clearing shrubs around roadways is part of land management in most national parks that aim to facilitate tourist experiences. Shrub removal practices are largely mechanical (although shrub cutting may be followed by herbicide treatment) and therefore clearing large areas and maintaining cleared areas remains challenging. Initiatives such as the development of transboundary reserves in the Lubombo Conservancy of Eswatini, could facilitate resource mobilization and facilitate wildlife and land management efforts. However, management may be most effective for biodiversity by promoting heterogeneity (e.g., McCleery et al. 2018), by balancing the proportion of areas with high shrub cover and cleared areas. Indeed, most of the bird species assessed here are associated positively with shrubs to some degree, indicating that shrubs may form critical resources for a diverse bird community within the ecosystem. Prioritizing the reduction of shrubs in highly encroached areas rather than wholesale removal of shrubs within mesic savannas would likely maximize biodiversity as shrubs form a crucial structural component of the mesic savanna.

## Author Contributions

**Muzi D. Sibiya:** writing – original draft (equal). **Wisdom M. Dlamini:** writing – original draft (equal). **Robert A. McCleery:** writing – original draft (equal). **Clelia Sirami:** writing – original draft (equal). **Ara Monadjem:** writing – original draft (equal). **Robert J. Fletcher Jr:** writing – original draft (equal).

## Conflicts of Interest

The authors declare no conflicts of interest.

## Supporting information


**Data S1:** ece372594‐sup‐0001‐supinfo.docx.

## Data Availability

The data and code that support the findings of this study are available for download on Dryad at https://doi.org/10.5061/dryad.08kprr5f4.
