# Supplementary material for: Twenty‐Two Years of Shrub Encroachment and Its Effects on Bird Communities in an African Savanna
Source: Ecol Evol. 2025 Dec 2;15(12):e72594. doi: 10.1002/ece3.72594 (PMC12671995; doi:10.1002/ece3.72594)
Supplement: Supplementary file 1 — Data S1: ece372594‐sup‐0001‐supinfo.docx. [file ECE3-15-e72594-s001.docx]

**SUPPORTING INFORMATION**

**Data Availability:**    <https://doi.org/10.5061/dryad.08kprr5f4>

**Table_1_Supp:** The total of 64 species that were recorded in ≥10% (≥14) of our plots in the low-lying savanna of Eswatini in 1998, 2008 and 2020. The 'encounter' column enumerates the total number of times a species was detected in the plots during the three periods. Also shown are species diet and habitat traits based on Hockey et al, 2005. Diet traits include invert=insectivore; seeds=granivore; nectar=nectar feeder and fruits=frugivore, whilst the nest column list nesting substrate predominantly utilized by the different species. The nesting trait 'para' indicates brood parasite behavior, where species lay their eggs in other species nests whereas Sav.woodland=savanna woodland, see Method section for further details.

| Species | Scientific name | Encounter | Diet | Nest | Habitat |
| --- | --- | --- | --- | --- | --- |
| African Paradise Flycatcher | *Terpsiphone viridis* | 40 | invert | tree | Sav.woodland |
| Barn Swallow | *Hirundo rustica* | 15 | invert | cavity | Grassland |
| Black backed Puffback | *Dryoscopus cubla* | 68 | invert | tree | Sav.woodland |
| Black collared Barbet | *Lybius torquatus* | 15 | fruits | cavity | Sav.woodland |
| Black crowned Tchagra | *Tchagra senegalus* | 50 | invert | shrub | Sav.woodland |
| Black Cuckooshrike | *Campephaga flava* | 45 | invert | tree | Sav.woodland |
| Black headed Oriole | *Oriolus larvatus* | 52 | invert | tree | Sav.woodland |
| Blue Waxbill | *Uraeginthus angolensis* | 64 | seeds | shrub | Sav.woodland |
| Brown crowned Tchagra | *Tchagra australis* | 56 | invert | shrub | Sav.woodland |
| Brown hooded Kingfisher | *Halcyon albiventris* | 14 | invert | cavity | Sav.woodland |
| Brubru | *Nilaus afer* | 66 | invert | tree | Sav.woodland |
| Cape Glossy Starling | *Lamprotornis nitens* | 48 | invert | cavity | Sav.woodland |
| Cape turtle Dove | *Streptopelia capicola* | 86 | seeds | tree | Sav.woodland |
| Cardinal Woodpecker | *Dendropicos fuscescens* | 49 | invert | cavity | Sav.woodland |
| Chinspot Batis | *Batis molitor* | 99 | invert | shrub | Sav.woodland |
| Common Scimitarbill | *Rhinopomastus cyanomelas* | 31 | invert | cavity | Sav.woodland |
| Crested Francolin | *Dendroperdix sephaena* | 17 | invert | grass | Sav.woodland |
| Dark capped Bulbul | *Pycnonotus tricolor* | 115 | invert | tree | Forest |
| Diederik Cuckoo | *Chrysococcyx caprius* | 18 | invert | para | Sav.woodland |
| Eastern Nicator | *Nicator gularis* | 51 | invert | shrub | Forest |
| Emerald spotted Wood Dove | *Turtur chalcospilos* | 99 | invert | tree | Sav.woodland |
| Fork tailed Drongo | *Dicrurus adsimilis* | 93 | invert | tree | Sav.woodland |
| Golden breasted Bunting | *Emberiza flaviventris* | 51 | seeds | shrub | Sav.woodland |
| Golden tailed Woodpecker | *Campethera abingoni* | 38 | invert | cavity | Sav.woodland |
| Gorgeous Bushshrike | *Telophorus viridis* | 14 | invert | shrub | Forest |
| Green backed Camaroptera | *Camaroptera brachyura* | 42 | invert | shrub | Forest |
| Green Wood Hoopoe | *Phoeniculus purpureus* | 18 | invert | cavity | Sav.woodland |
| Grey headed Bushshrike | *Malaconotus blanchoti* | 32 | invert | tree | Sav.woodland |
| Grey Tit Flycatcher | *Myioparus plumbeus* | 25 | invert | cavity | Sav.woodland |
| Helmeted Guineafowl | *Numida meleagris* | 17 | invert | grass | Sav.woodland |
| Klaass Cuckoo | *Chrysococcyx klaas* | 14 | invert | para | Sav.woodland |
| Kurrichane Thrush | *Turdus libonyanus* | 55 | invert | tree | Sav.woodland |
| Laughing Dove | *Streptopelia senegalensis* | 19 | invert | tree | Sav.woodland |
| Long billed Crombec | *Sylvietta rufescens* | 96 | invert | shrub | Sav.woodland |
| Marico Sunbird | *Cinnyris mariquensis* | 60 | nectar | tree | Sav.woodland |
| Orange breasted Bushshrike | *Telophorus sulfureopectus* | 51 | invert | tree | Sav.woodland |
| Pale Flycatcher | *Bradornis pallidus* | 18 | invert | tree | Sav.woodland |
| Purple banded Sunbird | *Cinnyris bifasciatus* | 19 | nectar | tree | Sav.woodland |
| Rattling Cisticola | *Cisticola chiniana* | 121 | invert | shrub | Sav.woodland |
| Red backed Shrike | *Lanius collurio* | 63 | invert | tree | Sav.woodland |
| Red billed Quelea | *Quelea quelea* | 17 | seeds | tree | Sav.woodland |
| Red chested Cuckoo | *Cuculus solitarius* | 18 | invert | para | Forest |
| Red eyed Dove | *Streptopelia semitorquata* | 29 | seeds | tree | Sav.woodland |
| Red faced Mousebird | *Urocolius indicus* | 41 | fruits | tree | Sav.woodland |
| Scarlet chested Sunbird | *Chalcomitra senegalensis* | 37 | nectar | tree | Sav.woodland |
| Sombre Greenbul | *Andropadus importunus* | 50 | fruits | shrub | Forest |
| Southern Black Flycatcher | *Melaenornis pammelaina* | 38 | invert | shrub | Sav.woodland |
| Southern Black Tit | *Parus niger* | 73 | invert | cavity | Sav.woodland |
| Southern Boubou | *Laniarius ferrugineus* | 23 | invert | shrub | Sav.woodland |
| Southern Grey headed Sparrow | *Passer diffusus* | 16 | seeds | cavity | Sav.woodland |
| Southern Yellow billed Hornbill | *Tockus leucomelas* | 17 | invert | cavity | Sav.woodland |
| Spotted Flycatcher | *Muscicapa striata* | 41 | invert | tree | Sav.woodland |
| Striped Kingfisher | *Halcyon chelicuti* | 24 | invert | cavity | Sav.woodland |
| Tawny flanked Prinia | *Prinia subflava* | 42 | invert | shrub | Sav.woodland |
| Violet backed Starling | *Cinnyricinclus leucogaster* | 37 | invert | cavity | Sav.woodland |
| White bellied Sunbird | *Cinnyris talatala* | 107 | nectar | shrub | Sav.woodland |
| White browed Scrub Robin | *Cercotrichas leucophrys* | 106 | invert | grass | Sav.woodland |
| White crested Helmet Shrike | *Prionops plumatus* | 28 | invert | tree | Sav.woodland |
| White winged Widowbird | *Euplectes albonotatus* | 23 | seeds | grass | Grassland |
| Willow Warbler | *Phylloscopus trochilus* | 17 | invert | tree | Sav.woodland |
| Woodland Kingfisher | *Halcyon senegalensis* | 63 | invert | cavity | Sav.woodland |
| Yellow breasted Apalis | *Apalis flavida* | 61 | invert | shrub | Sav.woodland |
| Yellow fronted Canary | *Crithagra mozambicus* | 60 | seeds | tree | Sav.woodland |
| Yellow throated Petronia | *Petronia superciliaris* | 67 | seeds | tree | Sav.woodland |

**Table_2_Supp:** Classification accuracy assessment for grass, shrub, and tree cover for the low-lying savanna of Eswatini in 1998, 2008 and 2020. For each year, class specific accuracy estimates are shown together with overall accuracy and the kappa coefficients.

| Year | Cover_class | User.acc (%) | Prod.acc (%) |
| --- | --- | --- | --- |
| 1998 | grass | 99.9 | 100 |
|  | shrub | 98.6 | 99.8 |
|  | tree | 99.9 | 99.9 |
| Overall accur. | 99.90% |  |  |
| Kappa.coeff. | 0.996 |  |  |
|  |  |  |  |
| 2008 | grass | 99.9 | 99 |
|  | shrub | 85.8 | 89.7 |
|  | tree | 96.6 | 96.6 |
| Overall accur. | 97.30% |  |  |
| Kappa.coeff. | 0.951 |  |  |
|  |  |  |  |
| 2020 | grass | 99.9 | 99.6 |
|  | shrub | 92.1 | 98.7 |
|  | tree | 99.9 | 99.9 |
| Overall accur. | 99.90% |  |  |
| Kappa.coeff. | 0.998 |  |  |


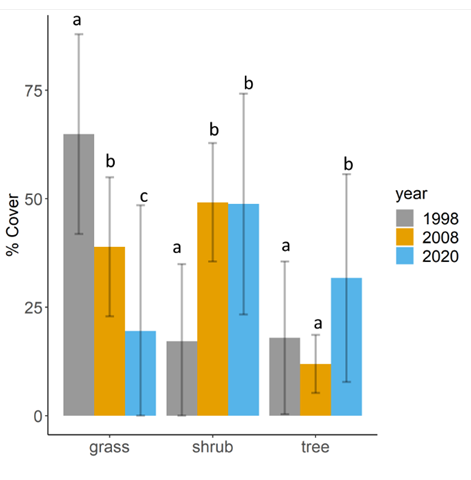


**Figure_1_Supp:** Overall change in the three vegetation cover types; grass, shrub and tree in the low-lying savanna of Eswatini as quantified in the wet season between 1998, 2008 and 2020 when we exclude five plots that were cleared of all woody vegetation between 2008 and 2020. The bar graphs indicate year sepcific everage cover and also shown are the standard errors (bars) for each cover type during each period. Letters, 'a', 'b' and 'c', represent pairwise comparison of mesaures with diffrent letters suggesting significant diffrences while shared letters indicate no difference. When excluding the five plots that were cleared of woody vegetation, we found that grass cover significantly declined during each period (1998, 2008 and 2020) whereas both variation in shrub and tree cover was similar to when we included all the plots.


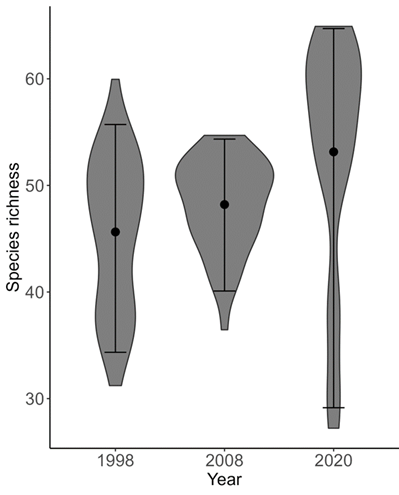


**Figure_2_Supp:** Estimated species richness (points = mean, error bars = 95% CRI), with violin plots showing posterior distribution for all (121) bird species counted in the low-lying savanna ecosystem of Eswatini during the wet seasons of 1998, 2008 and 2020.


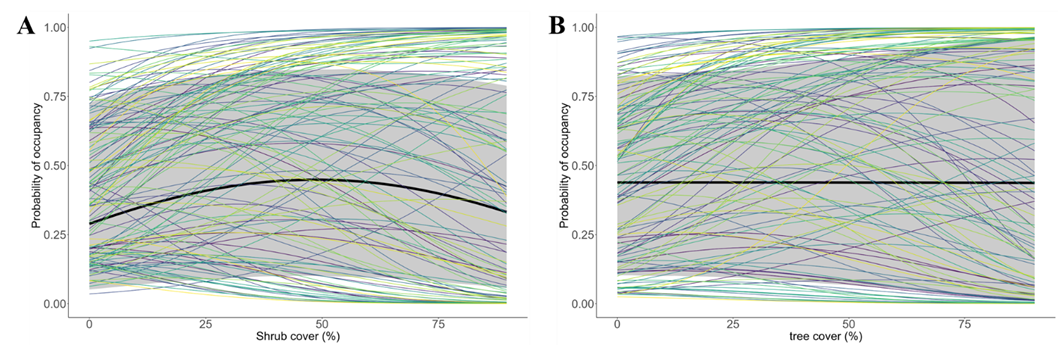


**Figure_3_Supp:** Community mean (thick black line) and species-specific occupancy (thin lines) responses to shrub (A) and tree (B) cover for all (121) bird species assessed in the low-lying savanna ecosystem of Eswatini during the wet seasons of 1998, 2008 and 2020. The grey ribbon delineates the 95% CRI for community responses.
